# Prevalence of Vitamin D Insufficiency and Its Determinants among Women Undergoing In Vitro Fertilization Treatment for Infertility in Sweden

**DOI:** 10.3390/nu15122820

**Published:** 2023-06-20

**Authors:** Paulina Maaherra Armstrong, Hanna Augustin, Linnea Bärebring, Amra Osmancevic, Maria Bullarbo, Ann Thurin-Kjellberg, Panagiotis Tsiartas

**Affiliations:** 1 Department of Obstetrics and Gynecology, Reproductive Medicine, Sahlgrenska University Hospital, 413 45 Gothenburg, Sweden; 2 Department of Internal Medicine and Clinical Nutrition, Sahlgrenska Academy, University of Gothenburg, 405 30 Gothenburg, Sweden; 3 Department of Dermatology and Venereology, Institute of Clinical Sciences, Sahlgrenska Academy, University of Gothenburg, 405 30 Gothenburg, Sweden; 4 Department of Dermatology and Venereology, Sahlgrenska University Hospital, 413 45 Gothenburg, Sweden; 5 Department of Obstetrics and Gynecology, Institute of Clinical Sciences, Sahlgrenska Academy, University of Gothenburg, 405 30 Gothenburg, Sweden; 6 Nordic IVF Solna, Eugin Group, 171 54 Solna, Sweden; 7 Department of Clinical Science, Intervention and Technology (CLINTEC), Division of Obstetrics and Gynecology, Karolinska Institute, 171 77 Stockholm, Sweden

**Keywords:** vitamin D insufficiency, 25-hydroxyvitamin D, infertility, in vitro fertilization, dietary assessment, sun exposure

## Abstract

There is a lack of research on women with infertility in the northern latitudes, where vitamin D insufficiency is high. Therefore, this study aimed to assess the prevalence and determinants of vitamin D insufficiency (serum 25(OH)D concentration < 50 nmol/L) among women undergoing in vitro fertilization (IVF) treatment. Thus, 265 women scheduled for IVF/intracytoplasmic sperm injection (ICSI) between September 2020 and August 2021 at Sahlgrenska University Hospital in Gothenburg, Sweden, were included. Data on serum 25(OH)D concentration, vitamin D intake, and sun exposure were collected via questionnaires and blood samples. Approximately 27% of the women had 25(OH)D insufficiency, which was associated with longer infertility duration. The likelihood of insufficiency was higher among women from non-Nordic European countries (OR 2.92, 95% CI 1.03–8.26, adjusted *p* = 0.043), the Middle East (OR 9.90, 95% CI 3.32–29.41, adjusted *p* < 0.001), and Asia (OR 5.49, 95% CI 1.30–23.25, adjusted *p* = 0.020) than among women from Nordic countries. Women who did not use vitamin D supplements were more likely to have insufficiency compared with supplement users (OR 3.32, 95% CI 1.55–7.10, adjusted *p* = 0.002), and those who avoided sun exposure had higher odds of insufficiency compared to those who stayed “in the sun all the time” (OR 3.24, 95% CI 1.22–8.62, adjusted *p* = 0.018). Women with infertility in northern latitudes and those from non-Nordic countries who avoid sun exposure and do not take vitamin supplements have a higher prevalence of 25(OH)D insufficiency and longer infertility duration.

## 1. Introduction

Vitamin D is a liposoluble steroid hormone that plays crucial roles in various physiological processes. Its endocrine effects are mainly related to the regulation of calcium and phosphorus metabolism, which are essential for bone and skeletal health [1]. Several studies have also suggested that vitamin D is involved in pathological conditions, such as neurological and cardiovascular diseases, as well as cancer [2,3,4]. 

The primary source of vitamin D is cutaneous synthesis, which occurs when the skin is exposed to ultraviolet B (UVB) radiation from sunlight. It can also be obtained from natural (cod liver oil, egg, fatty fish) and fortified (milk, yogurt, cereals) dietary sources and supplements [5,6,7,8]. The European Food Safety Authority Panel on Dietetic Products, Nutrition and Allergies and the Institute of Medicine recommend a serum 25-hydroxyvitamin D (25(OH)D) concentration of at least 50 nmol/L (obtained from both cutaneous synthesis and dietary sources) to ensure good musculoskeletal health and reduce adverse pregnancy-related outcomes [6,7]. 

Previous studies have shown that the prevalence of 25(OH)D insufficiency (serum 25(OH)D concentration < 50 nmol/L) in Northern Europe ranges from 7% to 34% [9,10]. Since sunlight exposure is lower at higher latitudes, the generally adequate vitamin D status in Nordic countries is mainly due to vitamin supplements and dietary fortification [11,12]. However, vitamin D status among immigrants in Northern Europe is still poor, mainly due to lower consumption of fatty fish, vitamin supplements, and clothing habits that reduce sunlight exposure [13,14]. 

The association between vitamin D and human reproduction is supported by studies linking it to polycystic ovary syndrome and endometriosis, which can lead to infertility [15,16]. However, the relationship between 25(OH)D status and outcomes of in vitro fertilization (IVF)/intracytoplasmic sperm injection (ICSI) remains unclear. A potential mechanism through which vitamin D insufficiency may impact in vitro fertilization IVF/ICSI outcomes is by exerting a negative effect on endometrial receptivity. Previous research has indicated the presence of vitamin D receptors in the endometrium and myometrium, suggesting a potential role of vitamin D in endometrial function [17]. Studies have demonstrated that vitamin D can up-regulate the expression of HOXA10, a crucial protein involved in embryo implantation and fertility. HOXA10 expression increases significantly during the implantation period [18], and it has been shown that vitamin D binds to the vitamin D receptor in human endometrial stromal cells, thereby potentially influencing the expression of HOXA10 [19]. Additional evidence from human endometrial cell lines supports this hypothesis, as the enzyme 1-alpha-hydroxylase, responsible for the conversion of calcidiol into calcitriol, the bioactive form of vitamin D, is up-regulated in the human endometrial stromal cells during early pregnancy [20]. Therefore, it is biologically plausible that vitamin D levels may indirectly affect implantation rates in infertile women undergoing IVF/ICSI through its influence on endometrial receptivity. Some studies have suggested lower clinical pregnancy and implantation rates in women with poor 25(OH)D status [21,22], whereas other studies have shown no impaired pregnancy rates among women with insufficient 25(OH)D status [23,24]. A recent meta-analysis of 15 cohort studies indicated a higher live birth rate among women with sufficient 25(OH)D status than among those with deficient status [25]. However, since both 25(OH)D status and IVF treatment success are influenced by various parameters, such as age, underlying infertility cause, skin phototypes, country of origin, and body mass index, the independent effect of vitamin D on IVF outcomes remains to be fully elucidated and warrants further investigation. 

The current study aimed to address the dearth of literature on the status of 25(OH)D in women with infertility seeking IVF treatment in Nordic countries. These countries are characterized by minimal cutaneous synthesis of vitamin D during the winter season, thereby potentially contributing to an expected higher prevalence of 25(OH)D insufficiency among this population. The primary aim of this study was to examine the prevalence of 25(OH)D insufficiency, both overall and seasonally, and to identify the determinants associated with this insufficiency in this population. 

## 2. Materials and Methods

**Figure 1:**
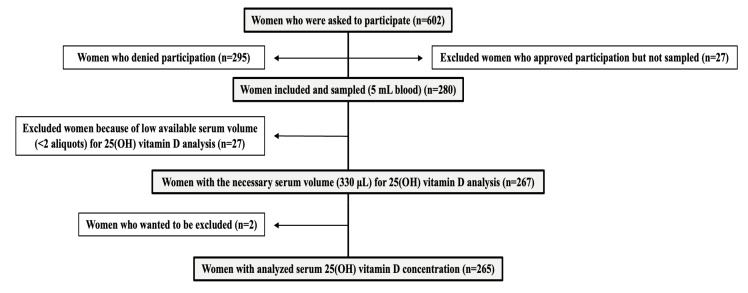
Flowchart of the study.

This prospective cohort study was conducted between September 2020 and August 2021 in the Department of Obstetrics and Gynecology, Reproductive Medicine, Sahlgrenska University Hospital, Gothenburg, Sweden. Gothenburg is a city located in southwestern Sweden (latitude 57.7° N), with a highly variable daylight duration between seasons. All women aged 18–38 years, who were scheduled for IVF/ICSI treatment during the study period, were invited to participate via mail within a couple of weeks prior to their planned visit to the clinics, and the recruitment of the participants was conducted continuously. To ensure the homogeneity of the study population receiving standard IVF/ICSI treatment and to minimize potential confounding factors, women undergoing pre-implantation genetic testing, sperm or oocyte donation treatments, and fertility preservation cycles were excluded from the study. This decision was motivated by several reasons. Firstly, excluding pre-implantation genetic testing was necessary to address concerns regarding the generalizability of findings, as the selection of embryos based on genetic criteria in this procedure could introduce biases not applicable to infertile women undergoing IVF/ICSI without such genetic interventions. Secondly, excluding participants undergoing sperm or oocyte donation treatments aimed to maintain methodological consistency, as these treatments involve distinct physiological and psychological aspects compared to standard IVF/ICSI cycles. Lastly, the exclusion of fertility preservation cycles was justified due to their specific protocols and medications, which could potentially influence the outcomes being studied. All invited women received a questionnaire validated for the dietary assessment of vitamin D intake, together with an invitation to participate in the study. The validation showed that the reported vitamin D intake, as assessed through the questionnaire, correlated well with both reported intake from 4-day food record and the biomarker 25(OH)D among pregnant women [26]. Written informed consent was obtained from all individual participants included in the study. The participants were asked to complete the questionnaire at home to minimize recall bias. Blood samples for the measurement of serum 25(OH)D concentration were collected in connection with participants’ first visit to the clinics upon consenting to participate. The number of women going through each step towards inclusion in the study and IVF/ICSI treatment is summarized in Figure 1.

Clinical data, including the woman’s age and BMI, smoking/snuffing habits, previous parity, antral follicle count, cause, and length of infertility, were obtained from the Reproductive Medicine electronic database. Data on country of origin, education level, skin phototype, UVB exposure (sun vacations, sun exposure habits), and dietary and supplemental intake of vitamin D were obtained from the questionnaires.

In this study, a venous blood sample (5 mL) was obtained from each participant to measure the serum 25(OH)D concentration. The blood samples were stored at room temperature and protected from light after collection. Within 8 h of collection, the samples were centrifuged at 2000× *g* for 10 min at room temperature, and the serum was stored at −80 °C until analysis. All frozen samples were analyzed in the same batch within 12 months of sampling after all participants were included. The analytical method used was Chemiluminescent Microparticle Immunoassay using 08P45 Alinity I, 25(OH) vitamin D reagent kit (Abbott Laboratories, North Chicago, IL, USA) and was performed at Clinical Chemistry, Sahlgrenska University Hospital in Gothenburg, Sweden. The analytical measurement uncertainty was 8% for a 25(OH)D concentration of 25 nmol/L and 6% for a concentration of 45 nmol/L. The detection level of 25(OH)D ranged from 9 to 771 nmol/L. For this study, vitamin D insufficiency was defined as a serum 25(OH)D concentration below 50 nmol/L, and a serum 25(OH)D concentration of 50 nmol/L or higher was considered sufficient.

The study questionnaire comprised demographic questions, including country of origin and education level, skin phototype, sun exposure habits, such as sun vacations, duration and type of sun exposure, and the size of sun-exposed body surface area. The country of origin was self-reported as Nordic or non-Nordic, with non-Nordic countries further categorized as non-Nordic European, Middle Eastern, African, Asian, and Central/South American. Education level was categorized as elementary school, high school, or university. Skin phototype was self-reported according to the Fitzpatrick scale as type I (always burn and never tan), type II (usually burn and tan less than average), type III (sometimes burn and tan), type IV (mildly burn and tan with ease more than average), type V (never burn and always tan more than average—brown), and type VI (never burn and tan more than average—black) [27]. Sun vacations were defined as travelling to a sunny country of low latitude (<35° N) for more than one week in the past four months before blood sampling. The duration of sun exposure was reported as hours of exposure on sunny days (less than one hour, between one and two, and more than two hours) and type of sun exposure as a preference for exposure on sunny days (in the sun all the time, both in the sun and shade, and in the shade all the time). The size of the sun-exposed body surface area was self-reported for face, hands, arms, legs, and core exposure (alone or in combination), and the percentage of sun-exposed regional body surface area (RBSA) was estimated by summing all the reported exposed single body areas (face: 7.5%, hands: 4.8%, arms: 14.9%, legs: 35.4%, core: 26.9%) [28]. Additionally, the questionnaire contained questions on vitamin D supplement use and consumption of food items (milk, fatty fish, yogurt/sour milk) that contribute to most of the vitamin D intake in Sweden. Daily dietary vitamin D intake was estimated as described by Bärebring et al. [26]. 

The primary objective of this study was to assess the prevalence (overall and seasonal) of vitamin D insufficiency and its determinants in women undergoing IVF/ICSI treatment. To assess the seasonal prevalence of 25(OH)D insufficiency, the cohort of women was subdivided into seasons based on the date of blood sampling, and seasons were defined according to the calendar definition of seasons for Sweden, each season lasting 3 months: spring, 1 March to 31 May; summer, 1 June to 31 August; autumn, 1 September to 30 November; and winter, 1 December to 28 or 29 February.

Descriptive statistics were used to report the frequency and percentage for categorical variables and as the median ± interquartile range (min-max) for continuous variables. To assess the univariate relationships between 25(OH)D status and continuous variables, the Mann–Whitney U test was employed, while Pearson’s chi-square test was used for the analysis of categorical variables. Pairwise logistic regression analysis was used to evaluate the differences between statistically significant multi-categorical factors obtained from the univariate chi-square analysis, and Bonferroni correction was applied for multiple tests. Unadjusted analyses were used for all demographic and outcome variables. Potential determinants (including season of blood sampling, country of origin, use of vitamin D supplements, daily dietary vitamin D intake, skin phototypes, type of sun exposure on sunny days, sun-exposed RBSA, age, BMI, and smoking) of the 25(OH)D status were first evaluated using univariate analyses. Significant prognostic factors were subsequently included in the multiple logistic regression model to assess independent and statistically significant factors for 25(OH)D insufficiency. Statistical significance was set at *p* < 0.05, and all analyses were performed using SPSS 28.0.1.0 for Mac.

## 3. Results

### 3.1. Prevalence of Serum 25(OH)D Insufficiency

During the study period of 2020–2021, 265 women consented to participate in the study, completed a questionnaire, and their blood was analyzed (Figure 1). Table 1 summarizes the demographic characteristics. The overall prevalence of 25(OH)D insufficiency was 27.2%, with a range of 7.4% in summer and 32.5% in winter. Women with 25(OH)D insufficiency had a significantly longer duration of infertility than those with sufficiency (median: 36 months vs. 24 months). Moreover, the prevalence of 25(OH)D insufficiency varied significantly depending on the participants’ country of origin. Specifically, women originating from Nordic countries had the lowest prevalence, whereas those from non-Nordic European, Middle Eastern, and Asian countries had a higher prevalence. Only a small number of participants originated from Africa (*n* = 3) and Central/South America (*n* = 2); therefore, they were excluded from the analyses. Furthermore, the use of vitamin D supplements and skin phototypes also showed significant differences in participants’ vitamin D status. Additionally, participants who reported being “in the shade all the time” had a significantly higher prevalence of 25(OH)D insufficiency compared to those who reported being “in the sun all the time” (42.1% vs. 15.4%). Women with 25(OH)D insufficiency also had smaller sun-exposed RBSA than those with sufficient levels (median: 82% vs. 89.5%).

### 3.2. Determinants of 25(OH)D Insufficiency

The determinants of 25(OH)D insufficiency were assessed using logistic regression analysis; the results are presented in Table 2. The country of origin (*p* < 0.001), use of vitamin D supplements (*p* = 0.002), and type of sun exposure on sunny days (*p* = 0.049) were identified as independent factors for 25(OH)D insufficiency. After adjusting for the season of blood sampling, sun-exposed RBSA, skin phototypes, age, BMI, and smoking, the odds for 25(OH)D insufficiency were higher for women originating from non-Nordic European countries (OR 2.92, 95% CI 1.03–8.26, adjusted *p* = 0.043), Middle Eastern countries (OR 9.90, 95% CI 3.32–29.41, adjusted *p* < 0.001), and Asian countries (OR 5.49, 95% CI 1.30–23.25, adjusted *p* = 0.020) than for women from Nordic countries. Furthermore, the odds for 25(OH)D insufficiency were higher for women who did not use vitamin D supplements compared to those who did (OR 3.32, 95% CI 1.55–7.10, adjusted *p* = 0.002). The odds for 25(OH)D insufficiency were also higher for women who reported staying “in the shade all the time” compared to those who reported staying “in the sun all the time” (OR 3.24, 95% CI 1.22–8.62, adjusted *p* = 0.018) but not for those who reported staying “both in the sun and shade” (OR 1.45, 95% CI 0.27–7.87, adjusted *p* = 0.664).

## 4. Discussion

This study represents the first investigation into the serum 25(OH)D status of infertile women residing at northern latitudes (57.7° N). Our main finding is an overall prevalence of serum 25(OH)D insufficiency of approximately 27%, with a seasonal prevalence ranging between 7% in summer and 32% in winter. Notably, the study identified country of origin, use of vitamin D supplements, and sun exposure as the main determinants of insufficiency. 

There are limited published data on the vitamin D status among women with infertility in Nordic countries. A previous study analyzing pooled data on serum 25(OH)D levels in 14 European countries found an overall prevalence of vitamin D insufficiency (<50 nmol/L) of approximately 40%, regardless of age group, ethnicity, or country latitude [9]. However, studies from Northern Europe, where sunlight exposure is limited due to high latitudes, have found a lower prevalence of vitamin D insufficiency compared to other European countries, ranging from approximately 7% to 34%, due to regular use of vitamin supplements, extended mandatory dietary fortification, and vacations in sunny locations [9,10,11,12]. A nationwide study of an unselected adult population in Sweden reported an overall prevalence of 25(OH)D insufficiency of 18% and 23% in the age groups 18–30 and 31–44 years, respectively. However, the small sample size of this study may limit the generalizability of its findings to the Swedish population [10]. In comparison, our study reported an overall prevalence of 25(OH)D insufficiency, which is consistent with studies from Northern Europe, albeit with a slightly higher prevalence compared to an unselected Swedish population with similar demographic and dietary characteristics. 

Previous studies from Nordic countries have revealed that immigrants are highly likely to suffer from 25(OH)D insufficiency due to various factors, such as limited consumption of vitamin D-rich foods (e.g., fatty fish), infrequent use of vitamin supplements, and wearing traditional clothing that restricts skin exposure to sunlight [13,14]. This finding highlights the significance of origin from non-Nordic countries as a determinant and predictor of poor vitamin D status. Our study’s results align with these findings, as we observed that women from non-Nordic countries had a higher prevalence of 25(OH)D insufficiency than those from Nordic countries. 

Previous studies and reviews have identified a potential role of vitamin D in female infertility [15,16] and have shown that vitamin D insufficiency is associated with dietary and supplemental intake, sun exposure, skin phototype, and clothing habits [10,13]. Sun exposure and skin phototype are the primary factors affecting cutaneous synthesis and the overall vitamin D status in humans [5]. Studies have found a high prevalence of 25(OH)D insufficiency among dark-skinned ethnic subgroups living in European countries and in Sweden [9,13,14]. Our study confirms these findings, demonstrating a high prevalence of 25(OH)D insufficiency among women with darker skin types who stayed only in the shade during sunny days and had a limited sun-exposed body surface area. Moreover, our study identified staying only in the shade during sunny days as an important predictive factor for low serum 25(OH)D concentration. These findings are attributed to the higher melanin concentration of darker-skin phototypes, which lengthens the duration necessary for cutaneous vitamin D production [5,8] and the clothing habits of some ethnic groups where most of the body surface is covered, reducing sun exposure during sunny days. In Nordic countries, where exposure to sunlight is limited, vitamin D intake through food and supplements is essential for counteracting unsatisfactory cutaneous production. However, our study found no difference in daily dietary vitamin D intake between the two groups, which could be attributed to the extended mandatory fortification of some food products in Sweden (i.e., milk, fermented milk products, and spreads) [12]. Nevertheless, our study showed that women with 25(OH)D insufficiency, intake fewer vitamin D supplements than those with sufficiency. Our findings suggest that the use of vitamin D supplements is a significant predictor of serum 25(OH)D levels. This observation is consistent with a previous study that showed that the use of vitamin supplements was associated with higher serum 25(OH)D concentration [10]. 

In our study, access to infertility treatment services was equal for all women, as fertility treatments were provided equally in Sweden through a tax-funded healthcare system. The eligibility criteria for receiving infertility treatment among couples with infertility entail the absence of previous children and a female partner under the age of 40 years. Moreover, the causes of infertility and education levels were comparable between women with 25(OH)D insufficiency and sufficiency. Despite these similarities, we found that women with 25(OH)D insufficiency sought infertility treatment later than those with sufficiency did. We believe that a contributing factor to this discrepancy in infertility length may be that nearly 57% of women with vitamin D insufficiency originated from non-Nordic countries. The reasons for delayed infertility treatment seeking among women from non-Nordic countries are likely multifaceted, including a lack of appropriate information on how and when to access infertility services, cultural barriers to infertility treatment, longer attempts to conceive naturally before seeking treatment, and other factors. This agrees with previous studies suggesting that race and ethnicity may contribute to delayed infertility treatment seeking, even in countries with comprehensive insurance coverage for such services [29]. 

Despite the established role of vitamin D in human reproduction and infertility-related conditions, such as polycystic ovary syndrome and endometriosis, the association of vitamin D with IVF/ICSI outcomes remains unclear due to discrepancies in study results [15,16]. A recent meta-analysis of 15 cohort studies including 3711 women undergoing IVF/ICSI showed that women with vitamin D concentrations >50 nmol/L had better live birth rates than those with vitamin D deficiency (<50 nmol/L) [25]. Therefore, studies like ours that assess the prevalence of vitamin D insufficiency play a crucial role in advancing our understanding of this relationship. By providing prevalence estimates in populations of women with infertility, these studies enable power analyses and help design future research endeavors. The findings from such studies can guide the development of randomized controlled intervention trials or large cohorts with well-defined timing of vitamin D measurements during the IVF/ICSI cycle and pregnancy. Additionally, accounting for the main demographic and lifestyle determinants of vitamin D status in these studies is essential to obtain more accurate and comprehensive results.

Our study had several strengths. First, we investigated a wide range of potential determinants of vitamin D status, including detailed assessments of vitamin D intake from both diet and supplements, multiple estimates of sun exposure, and evaluation of seasonal variations in serum 25(OH)D concentration. This comprehensive approach has allowed for a more nuanced understanding of the factors associated with vitamin D insufficiency. Second, we used country of origin rather than nationality or ethnicity to describe morphological characteristics, such as skin color. This approach improved the accuracy of our analysis by avoiding the potential misclassification of individuals who may share the same nationality but belong to different ethnic groups or have different countries of origin. 

The utilization of a questionnaire as a data collection tool to assess dietary intake of vitamin D is both a strength and limitation of our study. On the one hand, the questionnaire has been validated to assess dietary vitamin D intake, which is a positive aspect of our methodology [26]. On the other hand, questionnaires possess inherent limitations, such as recall bias and low accuracy of the information provided by the participants. To mitigate these limitations, all participants were provided with a questionnaire to complete at home a couple of weeks prior to inclusion in our study. Despite these efforts, it is important to note that the questionnaire did not contain inquiries regarding sun protection factor use during sun exposure, which could be a potential limitation of our study. In conclusion, the use of a questionnaire to assess dietary intake of vitamin D has both strengths and limitations, and efforts have been made to minimize the impact of inherent limitations. Nonetheless, it is important to acknowledge the limitations of our methodology to ensure accurate interpretation of the results. Another limitation of our study is the underrepresentation of blood sampling during the summer season, when 25(OH)D insufficiency is the lowest. The number of IVF/ICSI treatments performed during the summer were lower than in other seasons because of summer vacation in Sweden, resulting in closed IVF clinics and limited resources during the summer months. Balanced sampling throughout the year would have provided a more precise measurement of the seasonal prevalence of 25(OH)D insufficiency.

## 5. Conclusions

In this study, it was observed that three out of ten women with infertility residing in the northern latitudes had 25(OH)D insufficiency, with a higher incidence in the winter season. Notably, women with 25(OH)D insufficiency exhibited a longer duration of infertility as they initiated treatment later than women with sufficiency. The main determinants of 25(OH)D insufficiency are non-Nordic origin, lack of vitamin D supplementation, and limited exposure to sunlight. The study findings have important clinical implications, suggesting that addressing vitamin D insufficiency should be considered in the management of infertility, particularly among non-Nordic women. Strategies, such as vitamin D supplementation and tailored sunlight exposure, may improve vitamin D status and potentially influence IVF outcomes. However, further research is warranted to explore targeted interventions and elucidate the underlying mechanisms connecting vitamin D insufficiency and impaired fertility, enabling the development of novel therapeutic approaches.

## Figures and Tables

**Table 1:** Demographic characteristics of women sampled for serum 25-hydroxyvitamin D (25(OH)D) measurement and scheduled for IVF/ICSI treatment between 2020 and 2021.

	25(OH)D Insufficiency (<50 nmol/L) (*n* = 72)	25(OH)D Sufficiency (≥50 nmol/L) (*n* = 193)	Crude *p*-Value Pairwise Analyses (*)
Age (years)	32 ± 7 (20–39)	32 ± 6 (23–39)	0.384
BMI (Kg/m^2^)	24.7 ± 6 (19–38)	23.5 ± 6.5 (17–35)	0.068
Season of blood sampling			0.021
Spring (29.8%)	17 (21.5%)	62 (78.5%)	
Summer (10.2%)	2 (7.4%)	25 (92.6%)	0.011 (summer vs. winter) *
Autumn (29.8%)	17 (21.5%)	62 (78.5%)	
Winter (29%)	25 (32.5%)	52 (67.5%)	
Serum 25(OH)D (nmol/L)	39 ± 15 (15–49)	70 ± 20 (50–141)	<0.001
Spring (21.5% with insufficiency)	37 ± 15 (15–49)	64 ± 19 (50–114)	<0.001
Summer (7.4% with insufficiency)	39 ± 0 (37–41)	75 ± 18 (52–113)	0.030
Autumn (21.5% with insufficiency)	37 ± 19 (21–49)	73 ± 20 (51–136)	<0.001
Winter (32.5% with insufficiency)	42 ± 14 (19–49)	69.5 ± 19 (51–141)	<0.001
Previous pregnancies			0.958
Yes	24 (30.4%)	65 (69.6%)	
No	48 (27.3%)	128 (72.3%)	
Previous children			0.766
Yes	3 (20%)	12 (80%)	
No	69 (27.6%)	181 (72.4%)	
Infertility cause			0.889
Unexplained	2 (2.4%)	81 (97.6%)	
Male	21 (27.3%)	56 (72.3%)	
Anovulation	10 (25%)	30 (75%)	
Endometriosis	5 (35.7%)	9 (64.3%)	
Tubal	7 (33.3%)	14 (66.7%)	
Uterine	-	1 (100%)	
Premature ovarian insufficiency	1 (50%)	1 (50%)	
(missing data *n* = 1, 0.4%)			
Infertility duration (months)	36 ± 24 (17–120)	24 ± 12 (12–96)	<0.001
Mean antral follicle count	10 ± 7 (1–29)	9 ± 8 (1–30)	0.989
Country of origin			<0.001
Nordic	30 (16.5%)	152 (83.5%)	
Non-Nordic European	12 (41.4%)	17 (58.6%)	0.003 (Nordic vs. Non-Nordic European) *
Middle Eastern	19 (63.3%)	11 (36.7%)	<0.001 (Nordic vs. Middle East) *
Asian	8 (50%)	8 (50%)	0.003 (Nordic vs. Asian) *
African	1 (33.3%)	2 (66.7%)	
Central/South American	1 (50%)	1 (50%)	
(missing data *n* = 3, 1.1%)			
Education level			0.062
Elementary school	3 (37.5%)	5 (62.5%)	
High school	28 (35.4%)	51 (64.6%)	
University	38 (22%)	135 (78%)	
(missing data *n* = 5, 1.9%)			
Smoking/snuffing			0.584
No	65 (26.5%)	180 (73.5%)	
Yes	6 (33.3%)	12 (66.7%)	
(missing data *n* = 2, 0.8%)			
Use of vitamin D supplements			0.001
Yes	25 (18.3%)	112 (81.7%)	
No	47 (36.7%)	81 (63.3%)	
Daily dietary vitamin D intake (μg)	5.8 ± 6.8 (3.4–10.2)	6.9 ± 6 (4.2–10.2)	0.122
Skin phototypes (Fitzpatrick scale)			0.007
Type I	2 (28.6%)	5 (71.4%)	
Type II	23 (27.1%)	62 (72.9%)	0.004 (Type II vs. Type V) *
Type III	24 (22.2%)	84 (77.8%)	0.001 (Type III vs. Type V) *
Type IV	11 (27.5%)	29 (72.5%)	
Type V	11 (64.7%)	6 (35.3%)	
Type VI	1 (100%)	-	
(missing data *n* = 7, 2.6%)			
Sun vacations			0.649
Yes	2 (20%)	8 (80%)	
No	70 (27.5%)	185 (72.5%)	
Length of sun exposure on sunny days			0.109
Less than 1 h	15 (38.5%)	24 (61.5%)	
Between 1–2 h	25 (29.8%)	59 (70.2%)	
More than 2 h	31 (23%)	104 (77%)	
(missing data *n* = 7, 2.6%)			
Type of sun exposure on sunny days			0.022
In the sun all the time	10 (15.4%)	55 (84.6%)	
Both in the sun and shade	54 (30.7%)	122 (69.3%)	
In the shade all the time	8 (42.1%)	11 (57.9%)	0.016 (In the sun all the time vs. in the shade all the time) *
(missing data *n* = 5, 1.9%)			
Sun-exposed regional body surface area (%)	82 ± 62 (0–90)	89.5 ± 15 (0–90)	<0.001

IVF: in vitro fertilization; ICSI: intracytoplasmic sperm injection. The analysis of categorical variables was performed using the Pearson’s chi-square test or the Fisher’s exact test and was presented as number (*n*) and percentage (%) within the same variable. Continuous variables were analyzed using the Mann–Whitney U test and presented as median ± interquartile range and (min–max). When applicable, missing data was shown in a separate row. * indicates the statistically significant results from the pairwise logistic regression analysis that was used to evaluate the differences between multi-categorical factors.

**Table 2:** Logistic regression analysis for the assessment of the association between serum 25-hydroxyvitamin D insufficiency (<50 nmol/L) and potential determinants. Results were adjusted for confounding factors, such as the season of blood sampling, sun-exposed regional body surface area, skin phototypes, age, BMI, and smoking.

	Odds Ratio	95% CI	Adjusted *p*-Value
Country of origin			<0.001
Non-Nordic European vs. Nordic	2.92	1.03–8.26	0.043
Middle Eastern vs. Nordic	9.90	3.32–29.41	<0.001
Asian vs. Nordic	5.49	1.30–23.25	0.020
Use of vitamin D supplements	3.32	1.55–7.10	0.002
Type of sun exposure on sunny days			0.049
“In the shade all the time “ vs. “In the sun all the time”	3.24	1.22–8.62	0.018
“In the shade all the time “ vs. “Both in the sun and shade”	1.45	0.27–7.87	0.664

## Data Availability

The data presented in this study are available on request from the corresponding author.

## References

[1] Khazai N., Judd S.E., Tangpricha V. (2008). Calcium and vitamin D: Skeletal and extraskeletal health. Curr. Rheumatol. Rep..

[2] Moretti R., Morelli M.E., Caruso P. (2018). Vitamin D in Neurological Diseases: A Rationale for a Pathogenic Impact. Int. J. Mol. Sci..

[3] Gnagnarella P., Raimondi S., Aristarco V., Johansson H.A., Bellerba F., Corso F., Gandini S. (2020). Vitamin D Receptor Polymorphisms and Cancer. Adv. Exp. Med. Biol..

[4] Zhang R., Li B., Gao X., Tian R., Pan Y., Jiang Y., Gu H., Wang Y., Wang Y., Liu G. (2017). Serum 25-hydroxyvitamin D and the risk of cardiovascular disease: Dose-response meta-analysis of prospective studies. Am. J. Clin. Nutr..

[5] Lips P. (2006). Vitamin D physiology. Prog. Biophys. Mol. Biol..

[6] Ross A.C., Taylor C.L., Yaktine A.L., Del Valle H.B., Institute of Medicine Committee to Review Dietary Reference Intakes for Vitamin D., Calcium (2011). The National Academies Collection: Reports funded by National Institutes of Health. Dietary Reference Intakes for Calcium and Vitamin D.

[7] Bresson J.L., Burlingame B., Dean T., Fairweather-Tait S., Heinonen M., Hirsch-Ernst K.I., Mangelsdorf I., McArdle H., Naska A., Neuhäuser-Berthold M. (2016). EFSA NDA Panel (EFSA Panel on Dietetic Products, Nutrition and Allergies), 2016. Scientific opinion on dietary reference values for vitamin D. EFSA J..

[8] Neville J.J., Palmieri T., Young A.R. (2021). Physical Determinants of Vitamin D Photosynthesis: A Review. JBMR Plus.

[9] Cashman K.D., Dowling K.G., Škrabáková Z., Gonzalez-Gross M., Valtueña J., De Henauw S., Kiely M. (2016). Vitamin D deficiency in Europe: Pandemic?. Am. J. Clin. Nutr..

[10] Nälsén C., Becker W., Pearson M., Ridefelt P., Lindroos A.K., Kotova N., Mattisson I. (2020). Vitamin D status in children and adults in Sweden: Dietary intake and 25-hydroxyvitamin D concentrations in children aged 10–12 years and adults aged 18–80 years. J. Nutr. Sci..

[11] Jääskeläinen T., Itkonen S.T., Lundqvist A., Erkkola M., Koskela T., Lakkala K., Dowling K.G., Hull G.L., Kröger H., Karppinen J. (2017). The positive impact of general vitamin D food fortification policy on vitamin D status in a representative adult Finnish population: Evidence from an 11-y follow-up based on standardized 25-hydroxyvitamin D data. Am. J. Clin. Nutr..

[12] Itkonen S.T., Andersen R., Björk A.K., Konde Å.B., Eneroth H., Erkkola M., Holvik K., Madar A.A., Meyer H.E., Tetens I. (2021). Vitamin D status and current policies to achieve adequate vitamin D intake in the Nordic countries. Scand. J. Public Health.

[13] Granlund L., Ramnemark A., Andersson C., Lindkvist M., Fhärm E., Norberg M. (2016). Prevalence of vitamin D deficiency and its association with nutrition, travelling and clothing habits in an immigrant population in Northern Sweden. Eur. J. Clin. Nutr..

[14] Osmancevic A., Demeke T., Gillstedt M., Angesjö E., Sinclair H., Abd El-Gawad G., Landin-Wilhelmsen K. (2016). Vitamin D treatment in Somali women living in Sweden—Two randomized, placebo-controlled studies. Clin. Endocrinol..

[15] He C., Lin Z., Robb S.W., Ezeamama A.E. (2015). Serum Vitamin D Levels and Polycystic Ovary syndrome: A Systematic Review and Meta-Analysis. Nutrients.

[16] Somigliana E., Panina-Bordignon P., Murone S., Di Lucia P., Vercellini P., Vigano P. (2007). Vitamin D reserve is higher in women with endometriosis. Hum. Reprod..

[17] Vienonen A., Miettinen S., Bläuer M., Martikainen P.M., Tomás E., Heinonen P.K., Ylikomi T. (2004). Expression of nuclear receptors and cofactors in human endometrium and myometrium. J. Soc. Gynecol. Investig..

[18] Bagot C.N., Troy P.J., Taylor H.S. (2000). Alteration of maternal Hoxa10 expression by in vivo gene transfection affects implantation. Gene Ther..

[19] Du H., Daftary G.S., Lalwani S.I., Taylor H.S. (2005). Direct regulation of HOXA10 by 1,25-(OH)2D3 in human myelomonocytic cells and human endometrial stromal cells. Mol. Endocrinol..

[20] Viganò P., Lattuada D., Mangioni S., Ermellino L., Vignali M., Caporizzo E., Panina-Bordignon P., Besozzi M., Di Blasio A.M. (2006). Cycling and early pregnant endometrium as a site of regulated expression of the vitamin D system. J. Mol. Endocrinol..

[21] Paffoni A., Ferrari S., Viganò P., Pagliardini L., Papaleo E., Candiani M., Tirelli A., Fedele L., Somigliana E. (2014). Vitamin D Deficiency and Infertility: Insights From in vitro Fertilization Cycles. J. Clin. Endocrinol. Metab..

[22] Polyzos N.P., Anckaert E., Guzman L., Schiettecatte J., Van Landuyt L., Camus M., Smitz J., Tournaye H. (2014). Vitamin D deficiency and pregnancy rates in women undergoing single embryo, blastocyst stage, transfer (SET) for IVF/ICSI. Hum. Reprod..

[23] Banker M., Sorathiya D., Shah S. (2017). Vitamin D deficiency does not influence reproductive outcomes of IVF-ICSI: A study of oocyte donors and recipients. J. Hum. Reprod. Sci..

[24] van de Vijver A., Drakopoulos P., Van Landuyt L., Vaiarelli A., Blockeel C., Santos-Ribeiro S., Tournaye H., Polyzos N.P. (2016). Vitamin D deficiency and pregnancy rates following frozen–thawed embryo transfer: A prospective cohort study. Hum. Reprod..

[25] Iliuta F., Pijoan J.I., Lainz L., Exposito A., Matorras R. (2022). Women’s vitamin D levels and IVF results: A systematic review of the literature and meta-analysis, considering three categories of vitamin status (replete, insufficient and deficient). Hum. Fertility.

[26] Bärebring L., Amberntsson A., Winkvist A., Augustin H. (2018). Validation of Dietary Vitamin D Intake from Two Food Frequency Questionnaires, Using Food Records and the Biomarker 25-Hydroxyvitamin D among Pregnant Women. Nutrients.

[27] Fitzpatrick T.B. (1988). The validity and practicality of sun-reactive skin types I through VI. Arch Dermatol..

[28] Lee J.-Y., Choi J.-W. (2009). Estimation of Regional Body Surface Area Covered by Clothing. J. Hum.-Environ. Syst..

[29] Seifer D.B., Sharara F.I., Jain T. (2022). The Disparities in ART (DART) Hypothesis of Racial and Ethnic Disparities in Access and Outcomes of IVF Treatment in the USA. Reprod. Sci..

